# HLA-matched and HLA-haploidentical allogeneic CD19-directed chimeric antigen receptor T-cell infusions are feasible in relapsed or refractory B-cell acute lymphoblastic leukemia before hematopoietic stem cell transplantation

**DOI:** 10.1038/s41375-019-0610-x

**Published:** 2019-10-18

**Authors:** Xin Jin, Yaqing Cao, Luqiao Wang, Rui Sun, Lin Cheng, Xiaoyuan He, Xia Xiao, Yili Jiang, Qing Li, Huan Zhang, Wenyi Lu, Cuicui Lyu, Yanyu Jiang, Juanxia Meng, Mingfeng Zhao

**Affiliations:** 1 School of Medicine, Nankai University, Tianjin, 300071 China; 2 The First Central Clinical College, Tianjin Medical University, Tianjin, 300070 China; 3 Department of Hematology, Tianjin First Central Hospital, Tianjin, 300192 China

**Keywords:** Acute lymphocytic leukaemia, Immunotherapy

## To the Editor:

Chimeric antigen receptor-modified T (CAR-T) cells exhibit very effective function in elimination of relapsed/refractory B-cell acute lymphoblastic leukemia (R/R B-ALL) [1]. In patients with a high tumor burden or who have undergone heavy treatment, it is not always possible to manufacture an effective therapeutic product [2, 3]. Here, we describe the first-in-human use of HLA-matched allogeneic CAR-T cells (MCART19 cells) before allogeneic hematopoietic stem cell transplantation (allo-HSCT) and expand the description of HLA-haploidentical allogeneic CAR-T cell (HCART19 cell) infusion reported in previous sporadic cases.

Autologous CAR-T cells are difficult to produce due to high tumor burden or severe treatment. Eight patients with R/R B-ALL who had never received allo-HSCT received allogeneic CAR-T (allo-CART19) cell infusions. As of March 24, 2018, the median follow-up period was 10 months. All patients received more than four treatments before enrollment. One patient was PH positive (bcr-abl 190+), and seven were PH negative. Three patients had mutations associated with poor prognosis. All patients received lymphodepleting chemotherapy with fludarabine/cyclophosphamide. Except for patient 4, all patients exhibited a proportion of blast cells in the bone marrow that exceeded 80%. A median of 51.5% (range, 30–81%) of the infused T-cell mixture was CAR-T cells expressing anti-CD19. The patients numbered 1–4 received MCART19 cell infusions, and the other four patients received HCART19 cell infusions. The median infusion of allo-CART19 cells in the patients was 2 × 10^6^/kg (range, 5 × 10^5^–1 × 10^7^). Because infusion-related toxicity was mild, the four patients infused with the HCART19 cells (up to 1 × 10^7^/kg) received a higher dose than those infused with the MCART19 cells. All details are summarized in Tables 1 and S1.

**Table 1:** Infused cell characteristics and patient outcomes

Patient no.	CAR-T cell property	Anti-CD19 CAR-expressing T cells (%)	Anti-CD19 CAR-T-cell infusion/kg	Bone marrow evaluation	Survival after CAR-T-cell infusion	Clinical outcome	GVHD	CRS grade	Neurotoxicity grade	Interventions for infusion-related toxicities
1	HLA matched	70	5 × 10^5^	CR	4 months	Died of lung infection	No	3	2	Anti-allergy and antipyretic drugs, methylprednisolone, tocilizumab
2	HLA matched	40	5 × 10^5^	CR	14 months+	Has undergone subsequent allo-HSCT, sustained CR	No	3	3	Anti-allergy and antipyretic drugs, methylprednisolone, tocilizumab, plasma exchange
3	HLA matched	51	2 × 10^6^	PR	5 months+	Alive by supportive treatment	Uncertain	2	2	Anti-allergy and antipyretic drugs, methylprednisolone
4	HLA matched	44	2 × 10^6^	CR	3 months+	Has undergone subsequent allo-HSCT, sustained CR	No	2	3	Anti-allergy and antipyretic drugs, methylprednisolone
5	HLA haploidentical	52	2 × 10^6^	Morphologic residual leukemia	6 months	Died of disease progression	No	2	No	Anti-allergy and antipyretic drugs, methylprednisolone
6	HLA haploidentical	30	2 × 10^6^	Morphologic residual leukemia	8 months	Died of disease progression	No	1	No	No
7	HLA haploidentical	81	1 × 10^7^	Morphologic residual leukemia	1 month	Died of heart failure	No	2	No	Anti-allergy and antipyretic drugs, methylprednisolone
8	HLA haploidentical	80	1 × 10^7^	Morphologic residual leukemia	5 months	Died of disease progression	Uncertain	2	No	Anti-allergy and antipyretic drugs, methylprednisolone

*CAR* chimeric antigen receptor, *CR* complete response, *HLA* human leukocyte antigen, *Allo-HSCT* allogeneic hematopoietic stem cell transplantation, *GVHD* graft-versus-host disease, *CRS* cytokine response syndrome

All patients developed toxicity and required drug intervention after allo-CART19 cell infusion (Tables 1 and S2). Neurotoxicity and grade ≥ 3 cytokine release syndrome (CRS) occurred in three patients (patients 1, 2, and 4) treated with the MCART19 cells, and these patients developed hyperthermia, chills, muscle aches, apathy, loss of consciousness and cognitive disturbance accompanied by high levels of inflammatory cytokines and MCART19 cells in the peripheral blood (Figs. S1A–F and 1a). Under methylprednisolone treatment, anti-IL-6 antibody (tocilizumab) treatment, and other supportive treatments, patients 1 and 4 gradually recovered, but patient 2 deteriorated and had to be transferred to the intensive care unit to undergo plasma exchange. After three therapeutic plasma exchange cycles, patient 2 recovered consciousness, and both the fever and inflammatory indicators improved. We have reported the specific clinical course of patient in the form of a case report [4]. All patients developed fever after HCART19 cell infusion; nonetheless, in contrast to the MCART19 cell-treated patients, the HCART19 cell-treated patients experienced transient fever that could be controlled by antiallergy and antipyretic drugs. None of the four patients receiving the HCART19 cells developed neurotoxicity or grade ≥ 3 CRS. Patient 7 died of high tumor burden-related heart failure due to disease progression after 1 month of treatment with the HCART19 cells. The patient was observed to have a grade 2 CRS response, but no evidence of graft-versus-host disease (GVHD) and tumor lysis syndrome was found, so the patient was considered to be primarily dying of high tumor burden-related heart failure and was less likely to be associated with HCART19 cell infusion. Infusion-related toxicity in the other patients was controlled after an effective intervention. Patient 8 developed bloody stool due to a low platelet count before HCART19 cell infusion. After infusion, patient 8 again exhibited bloody stool, which was quickly relieved after supplementation with platelets. This adverse event was thought to be caused by the low platelet count rather than GVHD. Patients 3 and 8 experienced transient increases in their total bilirubin levels, and several patients developed elevated ALT, ALP, and GGT levels (Fig. S2A–E). Because these patients refused liver biopsy, it is not yet known whether these abnormal indicators were caused by GVHD-related liver damage. Other GVHD evidence, including rash and diarrhea, was not found. One study has reported that allo-CART19 cells within a certain density range have a dose-dependent killing effect on tumor cells but cause severe GVHD beyond this range. The absence of GVHD in our cohort may be because the patients were infused with the appropriate dose of allo-CART19 cells. Detailed adverse events are provided in Supplementary Table S2.

Three of the four patients (75%) who received the MCART19 cells (patients 1, 2, and 4) achieved complete response (CR) (Table 1). After MCART19 cell infusion, the white blood cell count of patient 1 stayed at a low level. One month after infusion, MCART19 cells became almost undetectable. Possibly due to immunocompromise, the patient developed an infection at 1 month after the infusion and eventually died of the severe infection. Patients 2 and 4 remained in remission for 14 months and 3 months after MCART19 cell therapy and subsequent allo-HSCT, respectively. The two patients received conditioning with total body irradiation, cyclophosphamide, antithymocyte globulin, fludarabine, and cytarabine after 2 months of MCART19 cell infusion, and infused HLA-haploidentical grafts from the younger brother and father, respectively. Two weeks after allo-HSCT, the chimerism monitored by short tandem repeats of these two patients were 96.37% and 97.51%, respectively, and both achieved complete donor chimerism (100% of donor alleles) at 1 month after allo-HSCT. Patient 3 was the only patient who underwent MCART19 cell infusion without achieving CR, but a significant reduction in his tumor burden was observed and assessed to be a partial response (PR) by bone marrow biopsies. The four patients treated with the HCART19 cells had a mild response to treatment, as assessed by monitoring the tumor burden in the bone marrow and extramedullary invasion, but relapsed shortly after therapy (Table 1). Patient 6 exhibited subcutaneous tissue infiltration in the right upper limb, and the infiltrating lesion was alleviated after HCART19 cell infusion. Three cases have been reported to have received HCART19 cells before allo-HSCT [5–7]. Two case reports have shown that the use of HCART19 cells as part of a conditioning regimen for allo-HSCT has beneficial therapeutic effects [6, 7]. In another case involving the HCART19 cells, no myeloablative conditioning regimen was used, and the patient had a mild response to treatment, which is consistent with the responses we observed [5]. Several reports have found that B-ALL relapses after treatment with autologous CAR-T cells. Even if the antigen recognized by CAR-T cells is still present, the disease will be resistant to a reinfusion of the same CAR-T cells [8–11]. The results for patients 3 and 5 suggest that this resistance cannot be removed even with allo-CART19 cells.

**Fig. 1:**
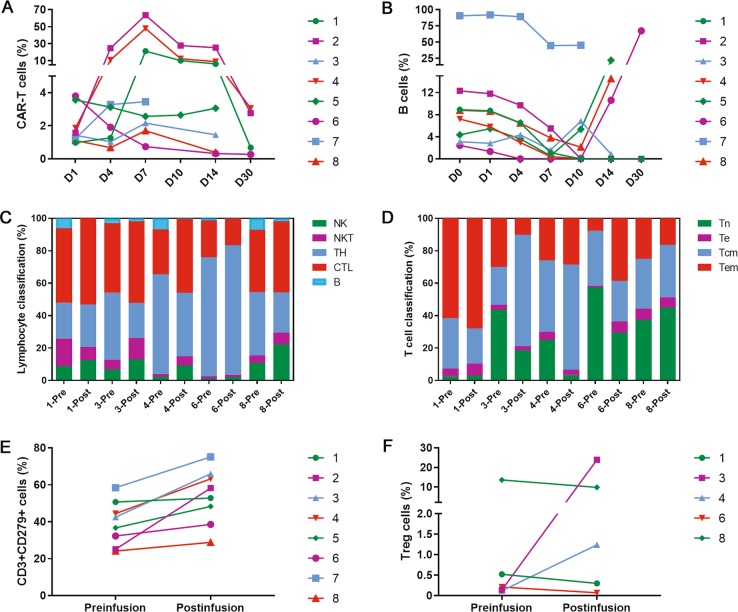
Allo-CART19 cell expansion, B-cell aplasia and lymphocyte phenotypic analysis occurred in the peripheral blood of patients. **a** The flow cytometry results showed that the proportion of MCART19 cells increased significantly in the peripheral blood of patients 1, 2, and 4. Compared with the other five patients, these three patients all had a good response to MCART19 cell therapy. **b** B-cell dysplasia occurred in patients 1, 2, and 4, who received MCART19 cell therapy. There was only a transient reduction in the proportion of peripheral blood B cells in the four patients treated with HCART19 cell therapy. **c** NK, NKT, B, TH, and CTL cells evaluated before and after cell infusion by flow cytometry. The number of NK cells in the five patient datasets available for review increased after infusion. **d** T cell phenotypic analysis before and after allo-CART19 cell infusion. Data for five patients were available and are displayed. Tn naive T cell, Te effector T cell, Tcm central memory T cell, Tem effector memory T cell. **e** Expression of PD-1 on T cells measured by flow cytometry before and after allogeneic CAR-T-cell infusion. All patients completed this evaluation, and almost all patients had increased PD-1 expression after infusion. **f** Treg cells measured by flow cytometry before and after allogeneic CAR-T-cell infusion. The proportion of Treg cells in patient 3 increased significantly after treatment

A high percentage of CAR-positive T cells in the total T-cell population was detected by flow cytometry in the three patients (patients 1, 2, and 4) who achieved CR after MCART19 cell treatment (Fig. 1a). In contrast, the proportion of CAR-positive T cells did not exceed 5% in the patients who did not achieve CR. Flow cytometry was performed to monitor the development of B cell aplasia by quantifying the number of CD19-positive B cells, and this methodology can be used as a pharmacodynamic measure of allo-CART19 cell function. For data reliability, we combined specific gene mutations, morphological smears, and CD22 antigen staining to exclude relapse with CD19 antigen loss of malignant B cells. B-cell aplasia occurred in the three patients (patients 1, 2, and 4) who all achieved CR after MCART19 cell treatment (Fig. 1b). There was only a transient reduction in peripheral blood B cell numbers in the patients who did not achieve CR. These results show that allo-CART19 cell infusion can specifically lyse CD19+ cells in vivo and that the level of CAR-positive T-cell expansion is directly related to the therapeutic response (Fig. 1a, b).

The difference in response to the MCART19 cells and HCART19 cells was assessed by analyzing serum cytokine levels, anti-CAR antibody levels, lymphocyte classifications (Table S3), and PD-1 expression on T cells before and after infusion. The results indicated that the NK cell frequencies of five patients increased after allo-CART19 cell infusion (Fig. 1c). The two evaluated patients who received the MCART19 cells and achieved CR had a higher percentage of cells with a Tem phenotype (Tem, CCR7-CD45RO+CD62L-CD45RA-) after infusion (Fig. 1d), and this result is similar to that of a previous study [12]. A detailed classification of the T cell phenotypes is provided in the Supplemental Materials (Fig. S3A, B). In all patients, PD-1 expression was higher on endogenous T cells than on allo-CART19 cells at the time of cell infusion (Fig. 1e). It is worth noting that the only patient who underwent MCART19 cell infusion and did not achieve CR had a significant increase in the proportion of regulatory T (Treg) cells and the expression of PD1 on T cells after infusion (Fig. 1e, f). We suspect that these increases are related to the patient's previous infusion of autologous CAR-T cells, but the exact mechanism remains unknown. One study also reported that PD-1 expression is increased after the infusion of allo-CART19 cells derived from posttransplant donors, which corroborates our results [13]. These data show that our allo-CART19 cells have properties similar to those of CAR-T cells derived from autologous or posttransplant donors. The lower efficacy of the HCART19 cells compared with that of the MCART19 cells may be due to the heterogeneity of the former in inducing graft rejection. It is unclear whether strengthening immunosuppressive treatment prior to infusion may enhance the anti-tumor effect of HCART19 cells, but this may also increase the risk of GVHD. However, this is only a hypothesis, and it still needs more evidence to be established.

Together, these cases suggest that allogeneic CAR-T cell therapy is feasible in R/R B-ALL and overcome limitation of autologous CAR-T cells, thus may be one possible regimen before the era of off-the-shelf “universal” CAR-T cell therapy. Compared with HCART19 cell infusion, MCART19 cell infusion had a higher CR rate and was accompanied by more severe toxic side effects. Although we did not observe GVHD or uncontrolled infusion-related toxicity, allo-CART19 cell therapy still requires close attention to GVHD and toxicity events. We have described only eight cases in a single center, and clinical trial studies to expand the sample size are urgently needed.

## Supplementary information

Revised-Supplementary Materials
