# Supplementary material for: HLA-matched and HLA-haploidentical allogeneic CD19-directed chimeric antigen receptor T-cell infusions are feasible in relapsed or refractory B-cell acute lymphoblastic leukemia before hematopoietic stem cell transplantation
Source: Leukemia. 2019 Oct 18;34(3):909–13. doi: 10.1038/s41375-019-0610-x (PMC7214243; doi:10.1038/s41375-019-0610-x)
Supplement: Supplementary file 1 — Revised-Supplementary Materials [file 41375_2019_610_MOESM1_ESM.docx]

Supplementary materials contain: supplementary methods, 3 supplementary tables and 3 supplementary figures.

Supplemental methods

*Clinical study design and protocol eligibility requirements*

This study was a clinical trial designed to assess the safety of infusing autologous or allogeneic T cells modified to express a CD19-specific CAR into patients with relapsed B-ALL (ChiCTR-ONN-16009862). We evaluated patients with autologous or allogeneic T cells based on their tumor burden, absolute lymphocyte count, and T cell function. The primary inclusion criteria for the patients treated with allo-CART19 cells were as follows: 1) peripheral blood lymphocyte counts below 0.2 × 10^9^/liter or peripheral blood with a high tumor burden (occupying more than 20% of the nucleated cells); 2) no receipt of allo-HSCT; 3) appropriate allogeneic (HLA-matched or HLA-haploidentical) T cells for preparing allo-CART19 cells; 4) diagnosis of CD19+ R/R B-ALL, where relapsed or refractory disease was deﬁned as no CR after more than two induction regimens or progression or recurrence of detectable disease after at least two cycles of salvage chemotherapy; and 5) an Eastern Cooperative Oncology Group performance status (ECOG PS) between 0 and 2. The primary endpoint of this study was the safety of the allo-CART19 cell infusion. Secondary endpoints included studies to detect allo-CART19 cell persistence as well as assays to assess morphology, cytology (assessed by flow cytometry), cytogenetics, and molecular antitumor responses. The the American Society for Transplantation and Cellular Therapy consensus grading for cytokine release syndrome and neurologic toxicity were used to grade CRS, neurologic events. The National Cancer Institute Common Terminology Criteria for Adverse Events version 5.0 was used to grade other adverse events. This study was approved by the Institutional Review Board of Tianjin First Center Hospital and was conducted in accordance with the Good Clinical Practice guidelines of the International Conference on Harmonisation. Informed consent was obtained from all patients after a discussion of the possible risks and adverse effects of the therapy.

*Assessment of clinical response*

The response to treatment was evaluated by bone marrow morphology and flow cytometry 4 weeks after the initial infusion. Using standard criteria, patient outcomes after allo-CART19 cell infusion were classified as CR, molecular CR, CR with incomplete platelet or neutrophil recovery (CRi), or morphologic residual disease. CR was defined as the disappearance of clinical and cellular evidence of leukemia. Hematopoiesis with a neutrophil count ≥1,000 × 10^6^/liter and a platelet count ≥100,000 × 10^6^/liter in the peripheral blood was considered normal. For CR, blasts accounted for <5% of the posttreatment bone marrow differential count. CRi was defined as meeting the criteria for CR but lacking adequate platelet or neutrophil recovery. Morphologic residual disease was defined as ≥5% blasts in the posttreatment bone marrow differential count.

*Production of CAR-T cells*

A single-chain variable fragment (scFv) sequence speciﬁc for CD19 was derived from the FMC63 monoclonal antibody. The CAR vectors contained the anti-CD19 scFv and human 4-1BB and CD3z signaling domains. CD3+ T cells were separated from peripheral blood mononuclear cells using CD3 immunomagnetic beads (#130-097-043, [Miltenyi Biotec](https://www.miltenyibiotec.com/), Germany) on day 1. The T cells were expanded using CD3/CD28 stimulating beads (#11131D, Thermo Fisher Scientific, USA) and IL-2 and were transduced with a lentiviral vector containing the anti-CD19 CAR gene construct on day 2. Then, the transduction efficiency was determined 5 days after transduction. Generally, these T cells were engineered via 10-12 days of manufacturing to express a CD19-specific CAR. CAR-T cells were released once they met the criteria for infusion. See the Supplemental Materials for other experimental methods.

*Detection of CAR expression by transduced T cells*

For each analyzed T cell culture, one sample of cells was stained with fluorescein isothiocyanate (FITC)-labeled polyclonal goat anti-mouse-F(ab)2 antibodies (#[115-096-006](https://www.jacksonimmuno.com/catalog/products/115-096-006), Jackson ImmunoResearch, USA) to detect anti-CD19 CAR-T cells. Subsequently, all cells were stained with phycoerythrin (PE)-labeled anti-CD3 antibodies (#[555340](http://www.bdbiosciences.com/cn/applications/research/t-cell-immunology/th-1-cells/surface-markers/human/pe-mouse-anti-human-cd3-hit3a/p/555340), BD Biosciences, USA).

*Assessment of toxic effects after CAR-T infusion*

Toxicities were evaluated relative to a baseline assessment conducted before CAR-T cell infusion. The concentrations of serum inflammatory markers including IL-2R, IL-6, IL-8, IL-10, TNF-α and C-reactive protein (CRP) were evaluated by Luminex assay, according to the manufacturer’s instructions.

*Immunophenotyping*

Anti-human monoclonal antibodies against CD3 (#552851, BD Biosciences, USA), CD4 (#555346, BD Biosciences, USA), CD8 (#557760, BD Biosciences, USA), CD16 (#561308, BD Biosciences, USA), CD19 (#555415, BD Biosciences, USA), CD25 (#560987, BD Biosciences, USA), CD45 (#560779, BD Biosciences, USA), CD45RO (#560608, BD Biosciences, USA), CD45RA (#561640, BD Biosciences, USA), CD56 (#561903, BD Biosciences, USA), CD62L (#561916, BD Biosciences, USA), CXCR3 (#550633, BD Biosciences, USA), CD127 (#561028, BD Biosciences, USA), CCR4 (#562579, BD Biosciences, USA), CCR6 (#560620, BD Biosciences, USA), CCR7 (#560765, BD Biosciences, USA), and CCR10 (#564771, BD Biosciences, USA) were used for immunophenotypic analysis. The specific labeled antibodies use for T cell phenotypic analysis are shown in Table S3. All flow cytometry analyses of stained cells were performed with a Coulter Altra flow cytometer equipped with CytExpert software (Beckman Coulter).

*Statistics*

Because of the small sample size of patients, the majority of this study is descriptive. Data were plotted using GraphPad Prism software version 7.0.

Supplemental tables

Table S1. Patient Characteristics

| **Patient no.** | **Age** | **Sex** | **ECOG PS** | **Diagnosis/ Cytogenetics** | **Initial therapy** | **Duration of CR1** | **No. of prior therapies** | **Marrow blasts before therapy (%)** | **Extramedullary invasion** | **Lymphodepletion before CAR-T cell infusion** |
| --- | --- | --- | --- | --- | --- | --- | --- | --- | --- | --- |
| 1 | 54 | F | 2 | t(9;22)(q34;q11) with TET2, PDGFRB and T315I mutations | VDCP+Imatinib | 48 weeks | 5 | 88.27 | No | FC |
| 2 | 23 | M | 2 | Normal karyotype with IKZF1 and TERT mutations | VDCLP | 62 weeks | 4 | 82.01 | Liver | FC |
| 3 | 17 | M | 1 | Normal karyotype | VDCLP | 2 months | 5 | 91.24 | No | FC |
| 4 | 23 | M | 1 | Normal karyotype with IKZF1 mutation | VDCLP | 2 months | 3 | 92.51 | No | FC |
| 5 | 21 | M | 2 | Normal karyotype | VDCLP | 30 weeks | 5 | 83.58 | No | FC |
| 6 | 17 | F | 1 | Normal karyotype | VDCLP | 28 weeks | 3 | Not found | Subcutaneous tissue | FC |
| 7 | 20 | M | 2 | Normal karyotype | VDCLP | 19 weeks | 7 | 93.12 | Pleural, stomach wall, kidney, subcutaneous tissue | FC |
| 8 | 18 | M | 2 | Normal karyotype | VDCLP | 52 weeks | 5 | 83.33 | Liver, spleen | FC |
| F: female; M: male; ECOG/PS: Eastern Cooperative Oncology Group/performance status; VDCP: vincristine, daunorubicin, cyclophosphamide, prednisone; VDCLP: vincristine, daunorubicin, cyclophosphamide, L-Asparaginase, prednisone; FC: fludarabine, cyclophosphamide; CR1: first complete response. | | | | | | | | | | |
|  |  |  |  |  |  |  |  |  |  |  |

Table S2. Observed adverse events related to chimeric antigen receptor (CAR)-T cell therapy

| **Adverse events** | **Grade 1** | **Grade 2** | **Grade 3** | **Grade 4** |
| --- | --- | --- | --- | --- |
| **Inflammation-related events** |  |  |  |  |
| Fever |  | 6 (135678) | 2 (24) |  |
| Febrile neutropenia |  | 8 (12345678) |  |  |
| Hypotension | 2 (78) | 4 (2345) |  |  |
| Cytokine release syndrome | 4 (5678) | 1（3） | 2 (14) | 1 (2) |
| **Hematological events** |  |  |  |  |
| Anemia |  | 1 (7) | 4 (1246) | 3 (358) |
| White blood cell decreased |  |  |  | 7 (1234568) |
| Neutrophil count decreased |  |  | 1 (4) | 7 (1235678) |
| Lymphocyte count decreased |  |  |  | 7 (1234568) |
| Platelet count decreased |  | 1 (4) |  | 7 (1235678) |
| Fibrinogen decreased |  | 2 (14) | 1 (2) |  |
| **Nervous system events** |  |  |  |  |
| Seizure | 1 (2) |  |  |  |
| Cognitive disturbance |  | 2 (14) | 1 (2) |  |
| Depressed level of consciousness |  | 2 (14) | 1 (2) |  |
| **Chemical laboratory abnormalities** |  |  |  |  |
| Alanine aminotransferase  increased | 4 (1236) | 1 (4) |  |  |
| Aspartate aminotransferase increased | 5 (12567) | 2 (34) |  |  |
| Gamma-glutamyltransferase increased | 2 (67) | 3 (258) | 2 (13) |  |
| Blood bilirubin increased | 1 (6) | 4 (1257) | 2 (34) |  |
| Creatinine increased | 1 (1) |  |  |  |

Grading according to the Common Terminology Criteria for Adverse Events version 5.0. The specific patient number is in parentheses.

Table S3. Labeled antibodies for T cell phenotypic analysis

| Non-T lymphocytes | B lymphocyte | CD3-CD19+ |
| --- | --- | --- |
|  | NK cell | CD3-CD16+/CD56+ |
| T lymphocyte subset frequencies | Total T lymphocyte population | CD3+ |
|  | Helper T cell | CD3+CD4+CD8- |
|  | Cytotoxic T cell | CD3+CD8+CD4- |
|  | NKT cell | CD3+CD16+/CD56+ |
| Th cell classification | Th1 cell | CD3+CD4+CXCR3+CCR4-CCR6-CCR10- |
|  | Th2 cell | CD3+CD4+CCR4+CCR6-CCR10-CXCR3- |
|  | Th17 cell | CD3+CD4+CCR4+CCR6+CCR10-CXCR3- |
|  | Regulatory T (Treg) cell | CD3+CD4+CD25+CD127dim |
| T lymphocyte classification (according to different developmental stages) | Naïve helper T cell | CD3+CD4+CCR7+CD45RA+CD62L+CD45RO- |
|  | Effector helper T cell | CD3+CD4+CCR7-CD45RA+CD62L-CD45RO- |
|  | Central memory helper T cell | CD3+CD4+CCR7+CD45RO+CD62L+CD45RA- |
|  | Effector memory helper T cell | CD3+CD4+CCR7-CD45RO+CD62L-CD45RA- |
|  | Naïve cytotoxic T cell | CD3+CD8+CCR7+CD45RA+CD62L+CD45RO- |
|  | Effector cytotoxic T cell | CD3+CD8+CCR7-CD45RA+CD62L-CD45RO- |
|  | Central memory cytotoxic T cell | CD3+CD8+CCR7+CD45RO+CD62L+CD45RA- |
|  | Effector memory cytotoxic T cell | CD3+CD8+CCR7-CD45RO+CD62L-CD45RA- |

Supplemental figures


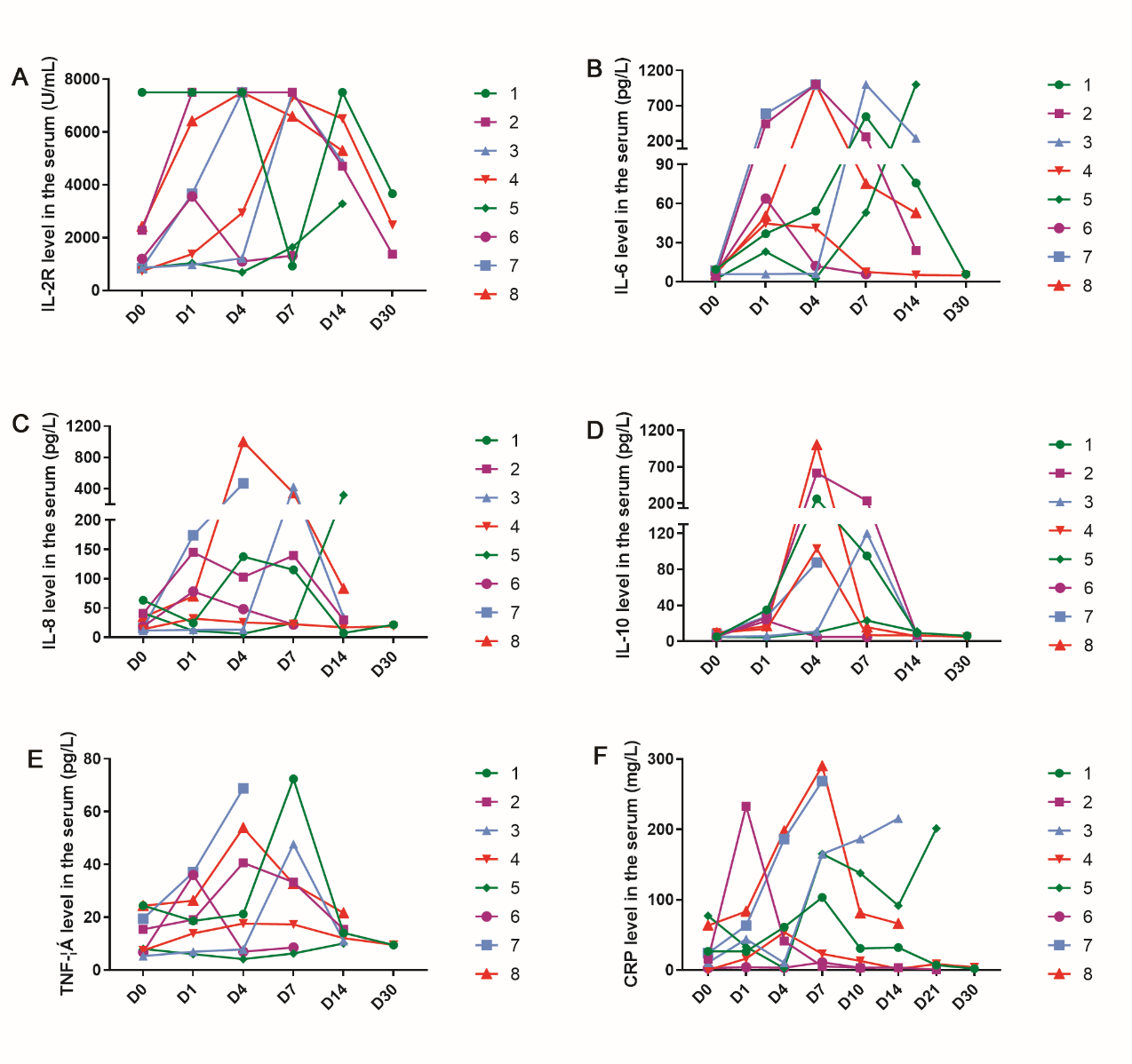


Fig. S1. Patients treated with MCART19 cells have higher levels of cytokines than patients treated with HCART19 cells. Patient serum cytokine levels were measured prior to and after allo-CART19 cell infusion by enzyme-linked immunosorbent assays (ELISAs). D0 represents 1 day before the infusion, and the monitoring lasted until 30 days after the infusion (D30). A to F show the dynamic serum levels of IL-2R, IL-6, IL-8, IL-10, TNF-α and CRP in 8 patients before and after infusion.


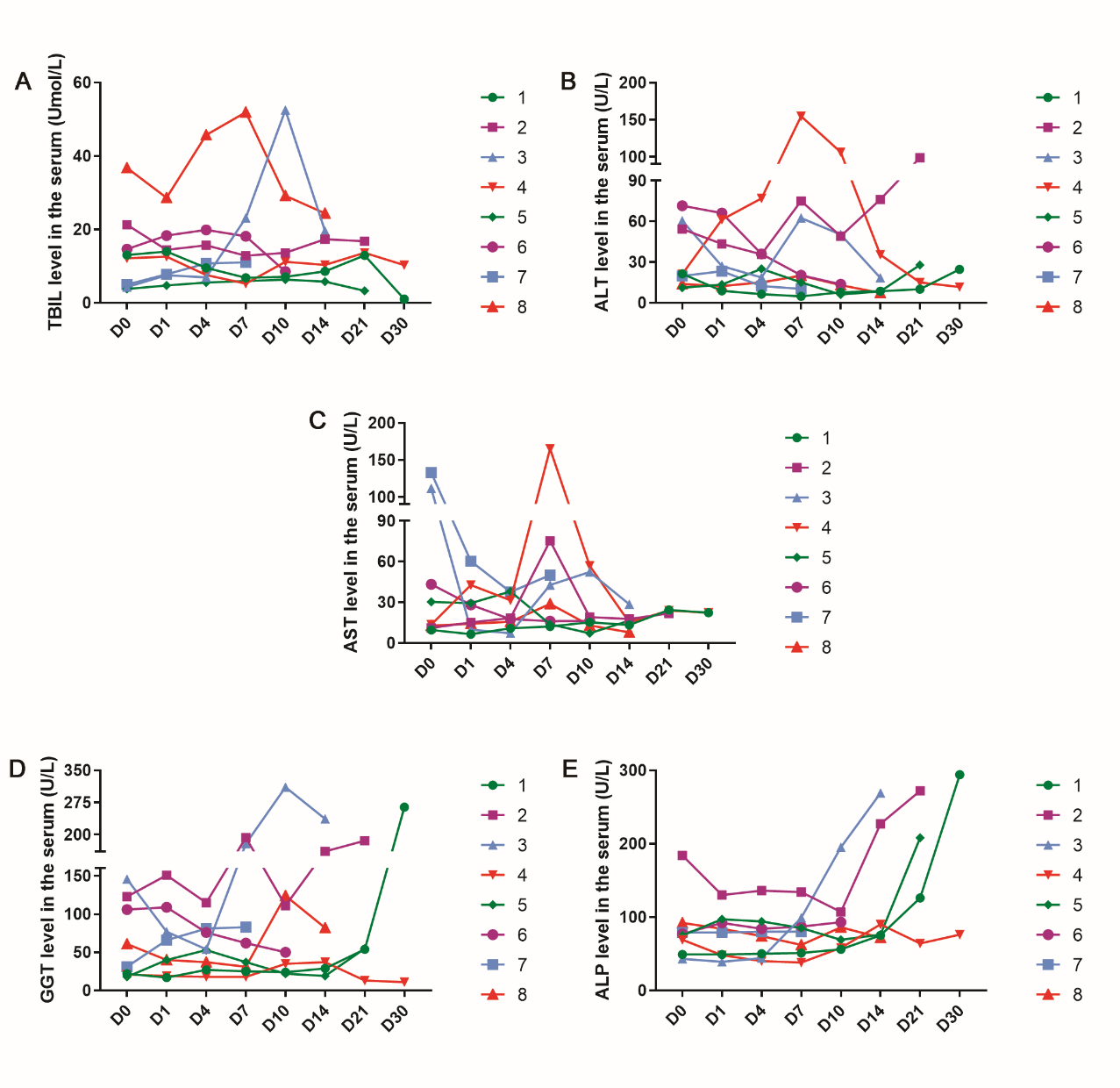


Fig. S2. There were several patients with transient increases in the levels of liver injury-related indicators. (A) Patient total bilirubin (TBIL) levels measured before and after allo-CART19 cell infusion. The levels of patient 3 and patient 8 peaked on Days 10 (D10) and 7 (D7), respectively, and quickly returned to normal levels. (B) Patient alanine aminotransferase (ALT) levels measured before and after allo-CART19 cell infusion. The levels of patient 4 rapidly decreased to normal after peaking, while those of patient 2 continually increased. (C) Patient aspartate aminotransferase (AST) levels measured before and after allo-CART19 cell infusion. (D) Patient gamma-glutamyltransferase (GGT) levels measured before and after allo-CART19 cell infusion. (E) Patient alkaline phosphatase (ALP) levels measured before and after allo-CART19 cell infusion.


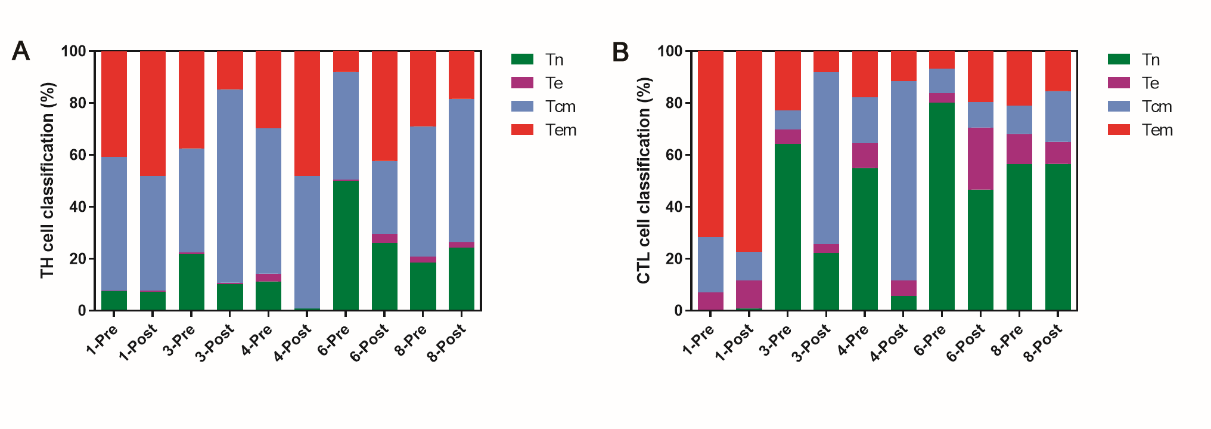


Fig. S3. Helper T (TH) cell and cytotoxic T lymphocyte (CTL)phenotypic analysis. (A) TH cell phenotypic analysis before and after allo-CART19 cell infusion. (B) CTL cell phenotypic analysis before and after allo-CART19 cell infusion. Tn, naive T cell; Te, effector T cell; Tcm, central memory T cell; Tem, effector memory T cell.
